# Evaluation of the In Vivo Anti-Atherosclerotic Activity of Quercetin Isolated from the Hairy Roots of *Hedysarum neglectum* Ledeb

**DOI:** 10.3390/life13081706

**Published:** 2023-08-08

**Authors:** Anna Vesnina, Irina Milentyeva, Varvara Minina, Oksana Kozlova, Lyudmila Asyakina

**Affiliations:** 1 Laboratory of Natural Nutraceuticals Biotesting, Research Department, Kemerovo State University, 650043 Kemerovo, Russia; irazumnikova@mail.ru; 2 Department of Genetic and Fundamental Medicine, Kemerovo State University, 650000 Kemerovo, Russia; vminina@mail.ru; 3 Department of Bionanotechnology, Kemerovo State University, 650043 Kemerovo, Russia; ms.okvk@mail.ru; 4 Laboratory of Phytoremediation of Technogenically Disturbed Ecosystems, Kemerovo State University, 650056 Kemerovo, Russia; alk_kem@mail.ru

**Keywords:** biologically active substances, plant materials, *Caenorhabditis elegans*, antioxidant activity, quercetin, hairy roots, *Hedysarum neglectum*

## Abstract

This study aimed to investigate the anti-atherosclerotic properties of quercetin isolated from the extract of *Hedysarum neglectum* Ledeb hairy roots. During the study, the hormonal composition of the nutrient medium for cultivation of *H. neglectum* hairy root biomass was selected: Gamborg’s medium enriched with the cytokine 6-benzylaminopurine (1.5 mg/1 dm^3^). It was found that the extraction of hairy root biomass with a 50% water–ethanol solution (40:1 1 h at 60 ± 2 °C) yielded an extract that contained the highest amount of quercetin (an average of 2.1 times higher than in extracts obtained at other parameters). It was determined that 100 µM quercetin solution showed the greatest bioactivity on *Caenorhabditis elegans*: on day 61, the percentage of surviving nematodes was 2.06 times higher compared to other samples and 6 times higher compared to control, resulting in a 12.5-fold increase in *SOD-3* expression compared to control (without biologically active substance (BAS) addition). Meanwhile, the 10 µM quercetin solution exhibited the best ability to inhibit the accumulation of lipid fractions; the accumulation was 1.06 times less compared to the control. The results of this study show that quercetin, which was isolated from the biomass of *H. neglectum* hairy roots, can be used as a component of anti-atherosclerotic dietary supplements.

## 1. Introduction

The average lifespan of the world’s population has increased significantly as a result of advancements in nutrition and medicine. Changes in the rhythms of life, environmental degradation, and the quality and quantity of a number of foods consumed by the population (fast food and highly processed foods) have resulted in the development of chronic diseases such as obesity, cardiovascular disease (CVD), degenerative diseases, gastrointestinal (GI) diseases, the metabolic syndrome, diabetes, and others [1,2].

Emphasis should be placed on the prevention of CVD since these diseases occupy a leading position in the structure of mortality in the world population, according to the World Health Organization [3]. Atherosclerosis is known to be the root cause of a number of CVDs, and one of the risk factors for its development is lifestyle, particularly nutrition. Dieting and the enrichment of the diet with products that exhibit cardioprotective activity, particularly anti-atherosclerotic activity, is thus an effective measure for the prevention of atherosclerosis and CVDs [4].

Details about the importance of nutrition in the prevention of atherosclerosis (diet, biologically active substances (BAS)–cardioprotectors) are reflected in the work of A. Vesnina et al. [5]. Therefore, adding foods high in biologically active substances (BAS) to the diet or taking dietary supplements (DS) containing these substances is a promising way to prevent the onset and progression of these diseases [6,7,8].

**Figure 1:**
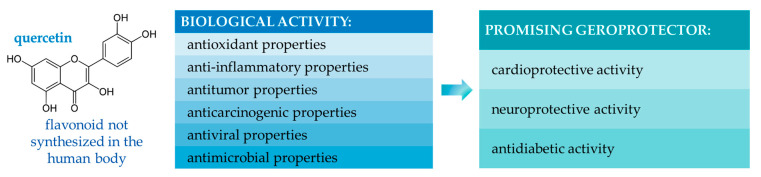
Biological activity of quercetin [11,12,13,14,15].

It is known that plants are a promising source of BAS–cardioprotectors [9,10] and that one of the potential cardioprotectors is quercetin, a flavonoid with extensive biofunctional effects [5,11,12,13,14,15] (Figure 1).

Quercetin exhibits anti-atherosclerotic activity. It is known that quercetin can inhibit the formation of reactive oxygen species, i.e., exhibit antioxidant activity. Quercetin can protect endothelial cells by inhibiting the formation of atherosclerotic plaques due to its effect on nitric oxide synthase. Quercetin can modulate lipid metabolism by inhibiting LDL oxidation, by influencing the HMG-CoA (5-hydroxy-3-methylglutaryl-coenzyme A) enzyme, by influencing reverse cholesterol transport. Quercetin exhibits anti-inflammatory activity by attenuating the NF-kB signaling pathway and by reducing the production of pro-inflammatory cytokines. It exhibits anti-apoptotic activity by promoting autophagy [16].

*Hedysarum neglectum* Ledeb. is a plant belonging to the Fabaceae family (consisting of about 300 species of annual and perennial herbs [17]). In folk medicine, it is known as “red root” and is used as a tonic, as an anti-inflammatory treatment, and for the treatment of gastrointestinal diseases, CVDs, and such. The pharmacological activity of this plant is due to the content of polysaccharides, flavonoids, catechins, tannins, alkaloids, etc. [18,19], which makes this plant a valuable raw material for the production of various dietary supplements. A study by L. Dyshlyuk et al. [19] proved the presence of quercetin in *H. neglectum* in amounts exceeding the content of other metabolites.

**Figure 2:**
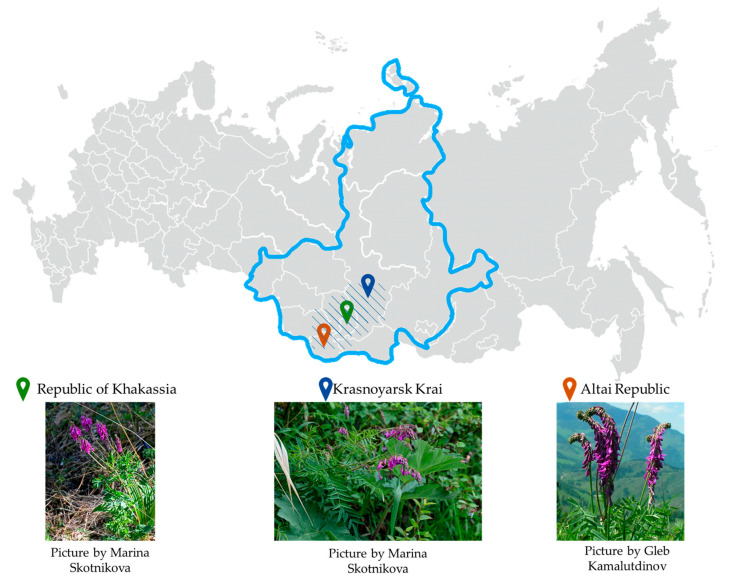
The geographic location of *H. neglectum* in Russia (shading reflects the plant distribution area, interpretation of information from https://www.plantarium.ru/page/view/item/18224.html, accessed on 4 May 2023).

This plant is popular in folk medicine in Russia, where it is sold as an officinal herb mixture, tea drink, dried root, crushed dried root, balm, and dry extract. In the Siberian Federal District, *H. neglectum* grows in high mountain, subalpine, and alpine zones; forest meadows; rocky hills; and thin larch forests, as shown in Figure 2 [20].

However, the natural resources of *H. neglectum* are insufficient to meet the population’s needs. As a result, it is preferable to use biotechnological cultivation methods, such as hairy root cultivation, to preserve biodiversity, the ecological situation, and the accumulation of sufficient amounts of plant raw materials for the extraction of BAS [21]. Thus, in vitro root cultures (hairy roots, transformed root cultures) of plants [22] are differentiated root cultures obtained by transforming a plant with the Gram-negative bacterium *Agrobacterium rhizogenes* (*Rhizobium rhizogenes*). Genetic transformation of plant cells occurs due to the introduction of transport DNA (Ri (root-induced) T-DNA) encoding *rolA*, *rolB*, *rolC*, and *rolD* genes into the plant genome by agrobacterium. These genes modulate auxin homeostasis, thereby causing the initiation and proliferation of hairy roots in vitro [23]. The advantages of using hairy roots include stable synthesis of secondary compounds, high growth rate [24,25], and genetic stability associated with greater differentiation of tissues and cells compared to callus and suspension cultures [26]. The ability of hairy roots to grow on hormonal nutrient media is a significant advantage; however, in order to accumulate the greatest amount of biomass and thus raw materials rich in metabolites, the authors of this study selected the hormonal composition of the nutrient medium to obtain the greatest amount of biomass in comparison to the control, the amount of biomass obtained by cultivation on nutrient media without hormones.

This study aimed to investigate the anti-atherosclerotic potential of quercetin isolated from the extract of *H. neglectum* hairy roots in order to further its use in/as a component of functional food supplements for preventive purposes.

The bioactivity of extracts from the biomass of *H. neglectum* hairy roots can be evaluated using a variety of model objects [5]. To date, it is known that the anti-atherosclerotic potential of quercetin was evaluated using various cell lines of rodents [16]. Nematodes are currently promising model organisms. *Caenorhabditis elegans* is a multicellular organism (a harmless free-living roundworm nematode) with several advantages [27,28]: (a) decoded genome, (b) similar to humans in behavioral and physiological health indicators (stress tolerance, degeneration of the nervous system, changes in muscle tissue structure), (c) small size (about 1 mm) and transparent body, (d) easy to cultivate and reproduce, and (e) short life cycle (about 3 weeks). 

*C. elegans* is known to be used to assess metabolic diseases in vivo. The model organism was used to assess the presence/absence of hyperglycemic effects through assessment of body fat accumulation [29,30] and to assess the antioxidant potential through assessment of gene expression of the body’s antioxidant defense system [31,32]. Therefore, *C. elegans* is a relevant model organism for assessing the anti-atherosclerotic activity of BAS. The aim of this research is to add to the body of knowledge regarding the anti-atherosclerotic potential of quercetin, which was isolated from an extract of the hairy roots of *H. neglectum* using *C. elegans*.

## 2. Materials and Methods

**Figure 3:**
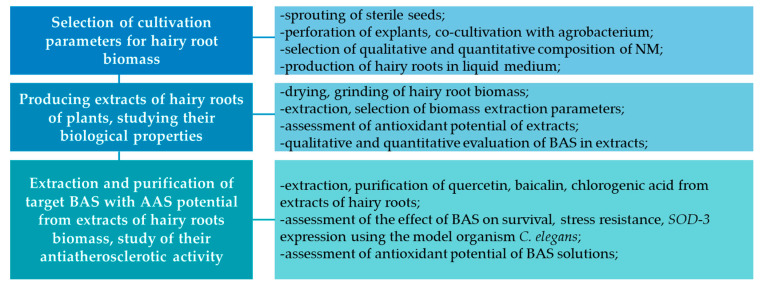
The main stages of implementation of the research objectives (AAS—anti-atherosclerotic).

The research was performed at KemSU Laboratory of Biotesting Natural Nutraceuticals. The flow diagram of the study is demonstrated in Figure 3.

One of the risk factors for chronic diseases such as atherosclerosis is oxidative stress. This study focused on protecting the nematodes from oxidative stress. Oxidative stress was induced by paraquat. In addition, the effect on the expression of the *SOD-3* gene, which encodes superoxide dismutase and is involved in cell antioxidant defense, was studied [33]. Additionally, since atherosclerosis is linked to impaired cholesterol metabolism, emphasis was placed on the ability to lower the amount of lipid inclusions in nematode bodies [34].

### 2.1. Cultivation of Hairy Roots

*H. neglectum* seeds purchased from Sady Rossii (Chibanovo village, Krasnoarmeisky district, Chelyabinsk region, Russia) were used to obtain hairy roots. Seeds were sterilized in 5 cm^3^ of solution (3% hydrogen peroxide and 96% ethanol in a 1:1 ratio) for 10 min at 25 ± 2 °C, then washed repeatedly with sterile distilled water and transferred for germination in Petri dishes on Murashige–Skoog solid medium (MS) [35] in dark conditions at 25 ± 0.5 °C for two days [35]. To produce hairy roots, 25–35 explants were used.

The seedlings were perforated with a sterile needle and incubated for 24 h in liquid MS medium containing a suspension of *Agrobacterium rhizogenes* ATCC 15834 [19]. A. rhizogenes bacteria were purchased from the collection of the Siberian Institute of Plant Physiology and Biochemistry, Siberian Branch of the Russian Academy of Sciences (Irkutsk, Russia).

The agrobacterial strains used in this study were grown on YEB medium for 48 h at 28 ± 0.5 °C [36]; a suspension of agrobacteria with an optical density of 0.8–1 determined at a wavelength of 600 nm was used to infect seedlings [37]. The composition of the YEB medium is presented in the article of G. Vervliet [38]. Spectrophotometric measurements of bacterial density were performed on a ClaioStar device (BMG, Offenburg, Germany).

After the indicated time, *H. neglectum* infected with agrobacteria were washed with sterile distilled water and placed on solid nutrient media, the composition of which is shown in Table 1, with the addition of 500 mg/dm^3^ of claforan (Thermo Fisher Scientific Inc., Bourgoin, France). Cultivation parameters: 25 ± 1 °C with 16.0 h photoperiod (cold white fluorescent lamps, illumination—2–3 klx). Experiments were transferred every three days until complete elimination of agrobacteria. Sterility of cultured roots was checked by incubating root samples in liquid YEB medium for 48 h at 26 ± 2 °C. The appearance of primary roots was observed 14–28 days after transformation.

Primary roots were separated and transplanted to agar medium with 250 mg/dm^3^ of claforan. The hairy roots were then transferred to antibiotic-free liquid medium and transplanted every three weeks by inoculating the crude root mass into conical flasks containing liquid nutrient medium [24,36,39]. Plant material was cultivated in the dark at 23 ± 0.5 °C on an orbital shaker (shaker incubator ES-20/80, BioSan, Riga, Latvia) at 90 rpm.

The compositions of the MS and B5 (Gamborg) media are presented in the articles by T. Murashige and F. Skoog [35] and O. L. Gamborg et al. [40], respectively. The literature review suggests that the ratios of inoculant to nutrient medium should be 1:100 and of flask volume to medium should be 5:1. When the hairy roots reached a size of 10–15 cm, they were separated from the plant and transferred to a new medium with the same components and subcultured every 30 days. The cultivation cycle was 5 weeks [24,36,39].

Explants punctured with a sterile needle but not infected with agrobacteria served as controls. Infected explants cultured on nutrient media without the addition of hormones were also used as controls. All experiments were performed in triplicate.

The difference in root dry mass at the end of cultivation and at the start of cultivation was used to calculate the growth index (I), which was calculated using Formula (1) [24]. Hairy root biomass was dried to constant weight in an air stream at 30 ± 0.5 °C in a desiccator (ShSvL-80—Kasimov (Kasimov priborniy zavod, Kasimov, Russia).

(1) $$I=\frac{X_{\max}-X_{0}}{X_{0}}\;,$$

where X_max_—mass at the end of the cultivation cycle, g;

X_0_—mass at the beginning of cultivation, g.

Appropriate nutrient media, indicated in Table 1, were used to maintain grown cultures.

Total genomic DNA was isolated from biosamples for molecular genetic analysis of the obtained hairy roots (to detect the integration of T-DNA plasmid Ri into the transformed roots (*rolB* gene). For DNA isolation, a commercial DNA-EXTRAN-3 kit (NPF Sintol, Moscow, Russia) was used. PCR analysis was performed on a TP4-PCR-01—Tertsik DNA amplifier (NPO DNK-Tekhnologiya, Moscow, Russia). Primers and specific amplification parameters for the *rolB* gene were previously reported by R. K. Tiwari et al. [37] and A. Stojakowska et al. [36] (Table 2).

Non-transformed DNA from the seedlings of the studied plants was used as a negative control for PCR analysis. PCR products (amplified DNA) were analyzed using electrophoretic separation on a 1% agarose gel and visualized in ultraviolet light after staining with ethidium bromide. Complex consisting of mixture A and B for amplification and primers was purchased from OOO NPF Sintol, Russia.

The biomass of hairy roots for further studies was accumulated by cultivating on liquid nutrient media, i.e., media of similar composition without the addition of agar–agar.

### 2.2. Preparation of Extracts from Hairy Roots Biomass, Evaluation of the Qualitative and Quantitative Composition of Metabolites

Filtration through a sterile paper filter (Yellow Ribbon GOST 12026-76 (Ekokhim, Novosibirsk, Russia) using a Buechner funnel under vacuum was used to separate the hairy roots from the culture liquid. Hairy root biomass was dried to constant weight in an air stream at 30 ± 0.5 °C in a desiccator (ShSvL-80—Kasimov (Kasimov priborniy zavod, Russia)). The dried biomass was ground in an LZM-1M mill (Olis, Barnaul, Russia) and sieved through a sieve with 1–3 mm apertures.

The plant material was extracted using a PE-4310 water bath (Ekroshim, Novosibirsk, Russia) with a reflux condenser. The extractant raw material was first exposed to ultrasound (US) for 15 min at a frequency of 40 kHz without allowing the water to heat above 40 °C, followed by extraction in a water bath with a reflux condenser. Ultrasonic (US) treatment was performed using a Stegler 3DT ultrasound bath (STEGLER, Taiwan, China) at an ultrasound frequency of 40 kHz. The extract was separated from the sediment of plant material using filtration with a sterile paper filter (Yellow Ribbon GOST 12026-76 (Ecohim, Saint Petersburg, Russia) and a Buechner funnel under vacuum. Table 3 presents the extraction parameters.

The mechanical effect of ultrasonic treatment promotes diffusion (penetration of the extractant into the cells of raw materials), destruction of cell walls, and accelerated release of their contents, which was discovered to be a relatively simple method of green chemistry. That is, when compared to the treatment without ultrasound, the method allows for a reduction in the duration and temperature of the BAS extraction process (without changing/increasing the yield of the extract) from plant raw materials [44,45].

A high-performance liquid chromatography (HPLC) method was used to determine the qualitative and quantitative content of polyphenols in extracts of hairy roots. The analysis was performed on a Shimadzu LC-20 Prominence chromatograph with a Shimadzu SPD20MA diode-matrix detector and a Phenomenex Gemini C-18 250 × 4.6 mm column (Shimadzu, Kyoto, Japan).

*H. neglectum* hairy root extracts were vacuum dried to a dry residue on an IKA RV 8 rotary evaporator (IKA^®^-Werke GmbH & Co. KG, Germany) at 45 ± 0.5 °C. Dry extract in the amount of 1 ± 0.05 g was added to 10 ± 0.01 cm^3^ of 1.5 M HCl solution. The resulting mixture was subjected to acid hydrolysis for 60.0 ± 0.5 min in a water bath with a reflux condenser (95 ± 5 °C). Then, the mixture was neutralized to pH = 7.0 by adding 1.0 M NaOH solution, filtered through a Yellow Ribbon GOST 12026-76 paper filter (Ecohim, Saint Petersburg, Russia), and then through a membrane filter with a pore size of 0.45 μm (Membrane Solutions, Auburn, WA, USA).

The gradient chromatography mode was used for all samples. Components of the mobile phase: acetonitrile, isopropyl alcohol, deionized water with the addition of orthophosphoric acid to pH = 3.5. Injection volume was 20 mm^3^, elution rate was 0.8 cm^3^/min, column temperature was 30 °C. The quantitative content was determined by the absolute graduation method using 98% purity standards: gallic acid, quercetin, protocatechic acid, chlorogenic acid, and caffeic acid (Sigma-Aldrich, Saint-Louis, MO, USA).

Quantitative analysis of the polyphenols under study was performed using calibration curves plotted in the concentration range of 1.9–235 µg/cm^3^. The equation for the calibration curves is presented in Formula (2).

(2) Y=a·X ,

where X—standard concentration (µg/cm^3^);

Y—corresponding peak area according to HPLC results;

a—factor of proportionality.

Formula (3) relates the peak area to a unit of dry mass.

(3) $$C=\frac{Y}{a\cdot m\cdot 1000}\;,$$

where C—flavone content in a sample of dry material (mg/g);

m—mass of dry material (g);

a—factor of proportionality from the equation of the calibration curve; factor 1000 is necessary to convert C to mg/g.

### 2.3. Extraction and Purification of Target Metabolites

**Figure 4:**
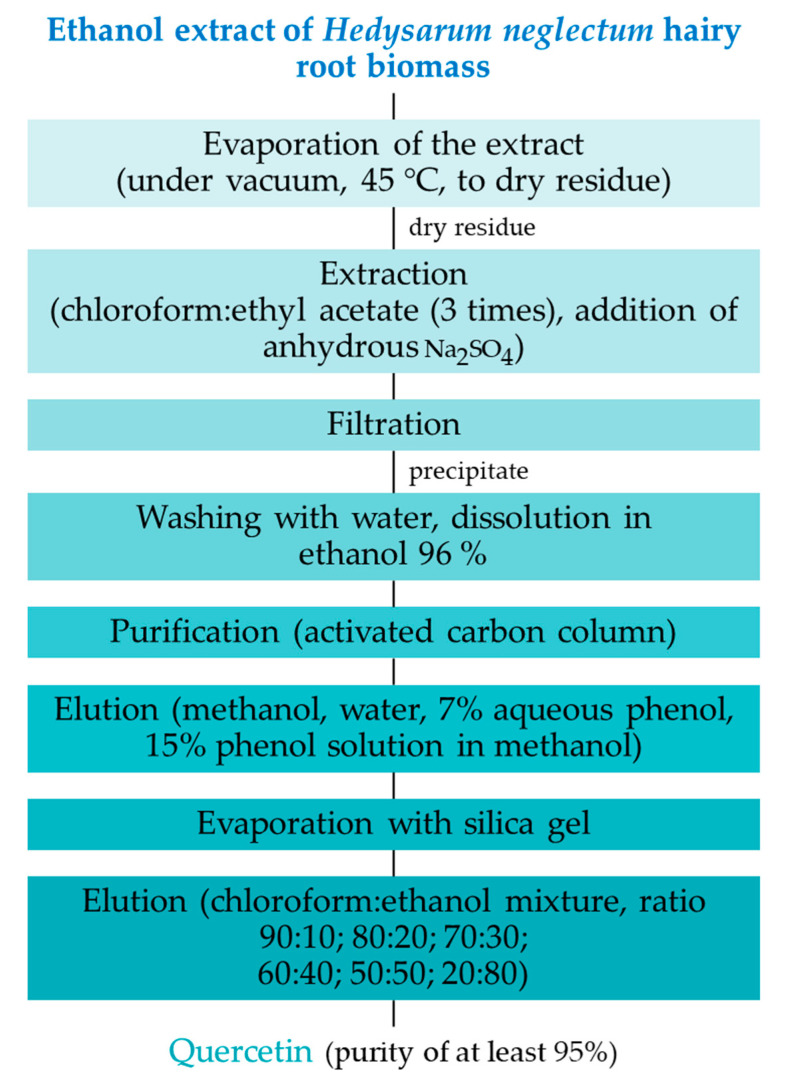
Scheme of extraction and purification of quercetin from *H. neglectum* hairy root biomass extract.

The dry residue on silica gel was placed on a 30 × 150 mm chromatographic column pre-packed with microcrystalline cellulose (Lachema, Karásek, Czech Republic) to isolate and purify the target BAS; elution was performed using a BioLogic low-pressure chromatograph (BioRad, Hercules, CA, USA). Preparative thin-layer chromatography (TLC) was performed to achieve a purity of at least 95%. Preparative TLC was performed on Sorbfil PTSKh-P-B plates (Labtex, Moscow, Russia). The extraction and purification of quercetin from hairy root biomass extracts is presented in the scheme shown in Figure 4.

An IKA RV 8 rotary evaporator (IKA^®^-Werke GmbH & Co. KG, Staufen, Germany), a sephadex LH-20 sorbent (GE Healthcare, Chicago, IL, USA), and activated carbon powder (Panreac, Barcelona, Spain) were used to evaporate the extracts.

The isolated BAS were identified using the following:

1. Spectrophotometric studies were performed on an SF-2000 spectrometer (OKB Spectr, Saint Petersburg, Russia); infrared spectroscopy was performed on an FSM-1202 device (Infraspect, Saint Petersburg, Russia). IR spectra were recorded in potassium bromide disks (Thermo Fisher Scientific Inc., Bourgoin, France). The results were processed using the FSpec 4.0.0.2 software. Spectra were recorded in the range of 4000–400 cm^−1^ with a resolution of 4 cm^−1^.
2. HPLC was combined with a mass spectrometric detector (HPLC-MS). The analysis was performed on a Waters Aquity UPLC chromatograph (Waters Corporation, Milford, CT, USA) equipped with an XEVO QTOF hybrid quadrupole time-of-flight mass spectrometer (Waters Corporation, Milford, CT, USA). A sample of 1 mm^3^ was applied to an ACQUITY UPLC BEH Phenyl column (50 × 2.1 mm, 1.7 μm; Waters, Drinagh, Ireland). The column temperature was 40 °C and the volume flow rate of the mobile phase was 0.4 cm^3^/min. A 0.1% (by volume) formic acid solution in water (solvent A) and a 0.1% (by volume) formic acid solution in acetonitrile (solvent B) were used as the mobile phase. The chromatographic separation of the substances was performed in the gradient elution mode. During the analysis, the composition of the mobile phase changed as follows (solvent B,% by volume): 0.0–1.0 min—15.0%; 1.0–5.0 min—15.0→30.0%; 5.0–15.0 min—30.0→38.0%; 15.0–15.5 min—38.0→45.0%; 15.5–23.0 min—45.0%; 23.0–23.5 min—45.0→95.0%. The analysis was performed in the positive ion detection mode (*m*/*z* range 100–1200). Ionization source parameters: ionization source temperature—120 °C, desolvation temperature—250 °C, capillary voltage—3 kV, sample introduction cone voltage—30 V, nitrogen feed rate (desolvation gas) 600 dm^3^/h. The results were processed LabSolutions version 5.73 software (Shimadzu, Kyoto, Japan).

### 2.4. Study of the Antioxidant Activity of Hairy Root Extracts and the Bioactivity of Quercetin Solutions

The antioxidant activity (AOA) of the obtained extracts and quercetin solutions was evaluated using the 2,2′-azino-bis (3-ethylbenzothiazoline-6-sulfonic acid) ABTS•+ cation radical decolorization method [46] on a UV 1800 spectrophotometer (Shimadzu, Japan):

1. Reagent ABTS (AppliChem GmbH, Darmstadt, Germany) was dissolved in water to a concentration of 7.0 mM (0.18011 g in 50 cm^3^ of water in a volumetric flask).
2. ABTS cation radical (ABTS•+) was obtained by reacting the stock solution of ABTS with 2.45 mM potassium persulfate (0.03311 g in 50 cm^3^ water in a measuring flask) and incubating the mixture in the dark at room temperature for 12–16 h before use. They were mixed in the ratio of ABTS:persulfate—2:1.
3. The ABTS•+ solution was diluted with ethanol to an optical density of 0.70 ± 0.02 at 734 nm.
4. To study the AOA, 3 cm^3^ of ABTS•+ was mixed with 30 mm^3^ of sample solution (100:1 ratio).

Control for ABTS•+ density determination—pure solvent. Control for samples—ABTS•+ without antioxidant. Formula (4) was used to calculate the AOA.

(4) $$AOA\;\left(\%\right)=\frac{A_{\mathrm{ABTS}}-A_{x}}{A_{\mathrm{ABTS}}}\times 100\%\;,$$

where A_ABTS_—optical density of the initial ABTS solution;

A_x_—optical density of ABTS solution + test sample.

The obtained AOA results were compared to the activity of different concentrations of vitamin C solutions (control). The extract with the highest AOA was chosen based on the results, and the target BAS were isolated from it.

The BAS under study were dissolved in a 70% aqueous–ethanol solution to obtain a 1 M stock solution [47], and solutions with concentrations of 800 μM, 600 μM, 400 μM, and 200 μM were prepared by dilution in water.

The bioactivity of the studied BAS isolated from extracts of plant hairy roots was tested on model organisms of *C. elegans* nematodes. The *C. elegans* N2 Bristol strain was provided by the Laboratory of Innovative Drug Development and Agrobiotechnology of the Moscow Institute of Physics and Technology (National Research University, Russia).

For the study, stock solutions of BAS in dimethyl sulfoxide (DMSO) at a concentration of 10 mM were prepared. Then, in distilled water, solutions with concentrations of 2000 μM, 1000 μM, 500 μM, and 100 μM were obtained. These solutions in an amount of 15 mm^3^ were added to the wells of the plate, thus obtaining test solutions of 200 µM, 100 µM, 50 µM, and 10 µM BAS concentration.

Pregnant adult nematodes were removed after 3 h of egg-laying on nematode growth medium (NGM) dishes in order to produce synchronized larvae by the age of 5–10. The dishes with eggs were treated with an aqueous NaCLO solution (salt:water ratio of 1:5). Eggs were cultivated on S medium at 20 ± 0.2 °C until hatching. The obtained synchronized L1-stage worms were placed on standard NGM agar dishes with *Escherichia coli* OP50 and incubated until the larval L4 stage [48]. The composition of the NGM and S medium is reflected in the work by T. Stiernagle [49].

*E. coli* strain OP50 was used as food for nematodes, provided by the V. Engelhardt Institute of Molecular Biology RAS (Russia). To obtain a starter culture of *E. coli* strain OP50, one colony was grown in 5 cm^3^ of LB medium (AppliChem GmbH, Germany) for 12 ± 0.5 h at 37 ± 0.5 ℃ under vigorous stirring (120 rpm).

Synchronized L4-stage larvae were transferred to NGM agar plates containing *E. coli* OP50 suspension at 20 ± 0.5 °C for 3 days. After the specified time, the larvae are in the L4 stage, to which 0.5 mg/cm^3^ of 5-fluoro-2-deoxyuridine (FUdR), a substance that inhibits nematode reproduction, is added, eliminating the possibility of obtaining a mixed worm population. After cultivating for 1 day at 20 ± 0.5 °C, the worms were transferred to liquid nutrient medium—S-medium. The experiments were performed in 96-well plates with a flat transparent bottom (Merck KGaA, Darmstadt, Germany), to which 150 mm^3^ of nematode and *E. coli* OP50 suspensions (bacteria concentration in the suspension: 0.5 mg/cm^3^) and 15 mm^3^ of different concentrations of tested BAS solutions were added [50,51].

To assess the life span of the nematode plates, they were cultivated at 20 ± 0.5 °C for 61 days. To assess the effect of BAS solutions on the stress tolerance of *C. elegans*, 15 mm^3^ of 1 M paraquat (a substance that simulates oxidative stress) were added to each well of the plate and the cultivation was continued at 20 ± 0.5 °C for 48 h in 6 h increments. After the specified time, live and dead nematodes were counted. Assessment was performed using an Axio Observer Z1 microscope (Karl Zeiss, Oberkochen, Germany). The criterion for the death of nematodes was the complete absence of motor activity when they were exposed to the bright light of the microscope beam. Nematodes suffering from internal hatching (embryos hatching inside an adult hermaphrodite) that escaped from the cups were censored (removed from the experiment). Experiments were performed in sterile conditions of a UVC/T-AR box (BioSan, Latvia). In more detail, the methodology of life span and stress tolerance assessment, the composition of nutrient media, etc., are presented in the paper by F. R. Amrit et al. [27].

A 24-well plate with 900 mm^3^ of L1-stage nematode suspension and 100 mm^3^ of BAS solution was used to evaluate the expression level of the *SOD-3* gene. The plate was left for 72 h at 20.0 ± 0.5 °C until the nematodes reached the L4 stage of development, and then the plate was placed in an incubator at 35 ± 0.5 °C for 5.0 h. After the specified time, the plate was cooled on ice and the content of the well was transferred to an Eppendorf tube and centrifuged for 2 min at 1000 *g* to remove the supernatant. The resulting precipitate was immediately frozen at 86 °C below zero until ribonucleic acid (RNA) was isolated. RNA was isolated from nematodes using the Exstract RNA kit according to the manufacturer’s instructions (Eurogen, Moscow, Russia). The quality of isolated RNA was assessed using a Nanodrop 2000C RNA concentration meter (Thermo Scientific, Waltham, MA, USA). Synthesis of the first cDNA chain was performed using the MMLV RT kit according to the manufacturer’s instructions (Eurogen, Russia). RNA amplification was performed on a CFX96 RealTime System (BioRad, USA) using a one-step approach with a 5X qPCRmix-HS SYBR reaction mixture designed for real-time PCR with SYBER Green I dye. The amplification steps are shown in Table 4.

The expression of the *SOD-3* gene in nematodes treated with the tested BAS solutions was compared to the expression of these genes in control samples—nematodes that had not been treated with the tested compounds. The quantitative gene expression levels were normalized relative to the expression levels of the AMA or ACT reference genes, which were determined using the same real-time amplification conditions and amount of cDNA tested.

Relative gene expression (RE) was calculated using Formula (5).

(5) $$\mathrm{RE}\;=E^{-∆∆\mathrm{Ct}},$$

where E—amplification efficiency (equal to 2.0).

Normalization by the mean of the reference genes is presented in Formula (6).

(6) $$∆C\left(t\right)=C{\left(t\right)}_{\mathrm{target}}-\;C{\left(t\right)}_{\mathrm{reference}\;},$$

where C(t)_reference_—geometric mean (C(t)) of reference genes (for mRNA);

C(t)_target_—C(t) of target genes.

The calculation of RE is presented in more detail in the paper by T.A. Dimitriadi et al. [52].

Fluorescent staining of lipid inclusions using the BDP 505/515 reagent bordipyrromethane fluorophore (Lumiprobe, Moscow, Russia) to visualize lipid membranes was used to assess the effect of BAS on fat accumulation in nematodes [34]. At the end of 10 days of nematode incubation in the presence of BAS, the contents of the 96-well flat-bottomed plate were transferred to the corresponding wells of the 96-well V-well plate. The plate was centrifuged at 1000 rpm for 3 min, and then the supernatant was removed without affecting the nematode precipitate. To wash the nematodes from the culture fluid, 150.0 mm^3^ of sterile PBS (8.0 g NaCl + 0.2 g KCl + 1.44 g Na_2_HPO_4_ + 0.24 g KH_2_PO_4_ dissolved in 1.0 dm^3^ water) were added to each cell and centrifuged at 1000 rpm for 3 min. The washing process was repeated twice. In each cell, 150 mm^3^ of fixative solution (40.0% isopropanol solution) were added to the washed nematode precipitate. The plates with nematodes were incubated for 20 min at 24.0 ± 2.0 °C. After this time, the plate was centrifuged at 1000 rpm for 3 min. Then, 150 mm^3^ of BDP 505/515 fat staining reagent working solution (2 mm^3^/cm^3^ of stock solution in 40% isopropanol) were added to each cell of the plate. The plate with nematodes was incubated for 15 min at 50 rpm 24.0 ± 2.0 °C. Then, it was centrifuged at 1000 rpm for 3 min and the supernatant was removed. The nematode precipitate was washed with sterile M9T solution (50 cm^3^ of M9 buffer (3 g KH_2_PO_4_ + 6 g Na_2_HPO_4_ + 5 g NaCl, autoclaving 20 min, 120 °C, then adding 1 cm^3^ of 1 M MgSO_4_ solution) with 5 mm^3^ Triton X-100) three times. After washing was completed, 100 mm^3^ of M9T were added to the wells and the stained lipid inclusions in the nematode bodies were scanned using an ImageXpress Mico XL automated fluorescence microscope (Molecular Devices, Silicon Valley, CA, USA) [34].

### 2.5. Statistics Section

Experiments were performed in triplicate. The results were expressed as mean ± standard deviation. In the tables and figures, values marked with an * are significantly different from the others (*p* < 0.05), according to the Tukey post hoc criterion score. Statistica 10.0 software (StatSoft, Inc., Tulsa, OK, USA) was used for the analysis.

## 3. Results

### 3.1. Hairy Root Cultivation Results

*H. neglectum* hairy roots were obtained during biotechnological cultivation on nutrient media (NM) with varying hormonal compositions; a description of hairy roots is provided in Table 5.

**Figure 5:**
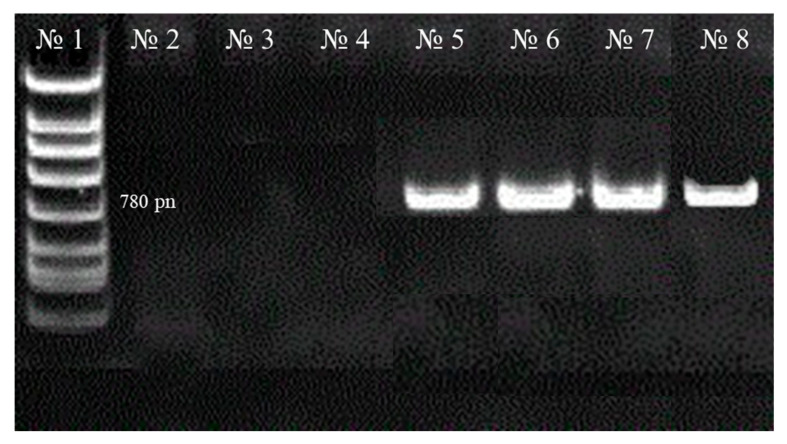
Results of electrophoresis of amplification products in agarose gel: No. 1—DNA marker; No. 2, 3, 4—control samples isolated from DNA of untransformed objects; No. 5, 6, 7—samples with rolB gene in the roots of different *H. neglectum* samples grown on nutrient medium No. 3. Following cultivation of *H. neglectum* hairy roots, it was discovered that all of the studied biobacteria were transformed by agrobacteria.

In control samples (non-transformed seedlings), the formation of hairy roots was absent, which was confirmed by PCR analysis for the presence of the *rol B* gene in the isolated DNA. The results of PCR analysis to determine *A. rhizogenes* contamination of bioobjects showed that 780 bp amplification products were detected in all tested DNA isolated from *H. neglectum* hairy root samples grown on nutrient media No. 3 (Figure 5).

Following cultivation of *H. neglectum* hairy roots, it was discovered that all of the studied biobacteria were transformed by agrobacteria and that it is best to use nutrient medium No. 3, which contains 1.5 mg of synthetic cytokine 6-BAP per 1 L of medium.

**Figure 6:**
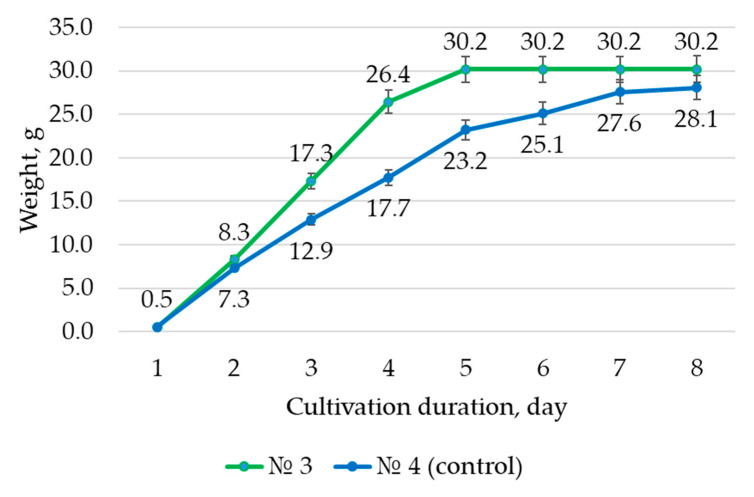
Growth curves of *H. neglectum* hairy roots.

Figure 6 demonstrates the results of the biomass accumulation of the *H. neglectum* hairy roots in liquid nutrient medium.

All growth curves were approximately S-shaped: The exponential growth phase began at 28 ± 1 days and the die-off phase at 35 ± 1 days. Thus, the maximum values of the growth index of hairy roots were obtained when growing on NM No. 3. Compared to the control sample (No. 4), the growth index was greater by 7.8%.

### 3.2. Preparation of Extracts from Hairy Root Biomass, Evaluation of the Qualitative and Quantitative Composition of Metabolites

Plant biomass was separated from the culture liquid, dried, and ground to obtain dry hairy root powder. An appropriate extractant was added to the dried biomaterial, and the mixture was subjected to ultrasonic treatment and extraction in a water bath with a reflux condenser. The extraction parameters of dried and ground biomaterial of hairy roots are presented in Table 6.

During the extraction of hairy roots, biomass samples were obtained, which are distinguished by the following characteristics: color—red brown, smell—typical of *H. neglectum* alcohol extract, and peculiarity—the presence of sediment.

**Figure 7:**
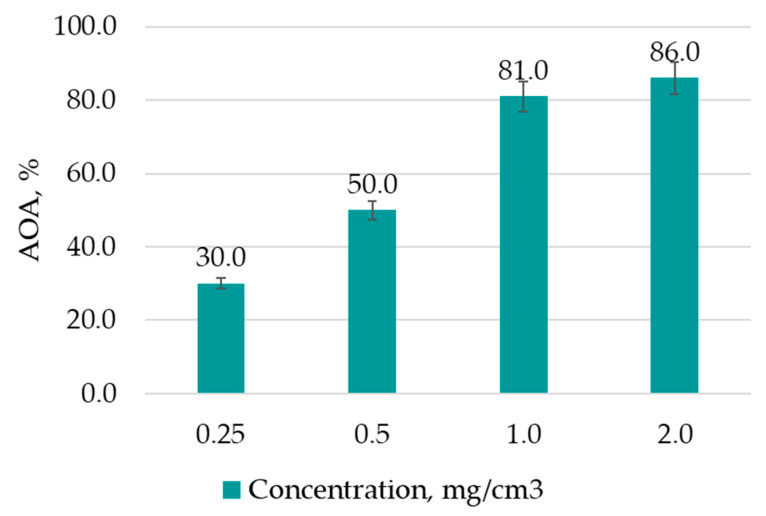
AOA of vitamin C (control) of different concentrations, %. Data are presented as mean value ± SD (n = 3).

The AOA of the extracts obtained from the biomass of hairy roots was evaluated to select the extraction parameters that would allow a sample containing the greatest amount of antioxidant substances to be obtained. The AOA results of the extracts obtained from hairy root biomass are presented in Table 7. For comparison, the AOA of vitamin C solutions of different concentrations was evaluated; the results are demonstrated in Figure 7.

All studied extracts exhibited an AOA exceeding the activity of vitamin C (2 mg/cm^3^) by 8.5% on average.

**Figure 8:**
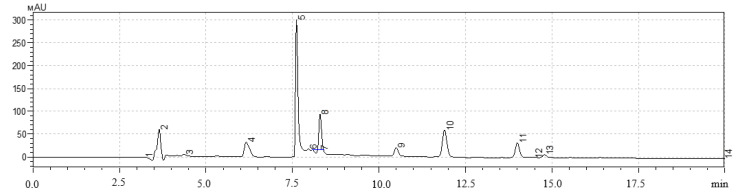
Chromatogram of extract No. 1 of *H. neglectum* hairy roots (300 nm): peak 5—gallic acid, 8—quercetin, 9—protocatechic acid, 10—chlorogenic acid, 11—caffeic acid.

**Figure 9:**
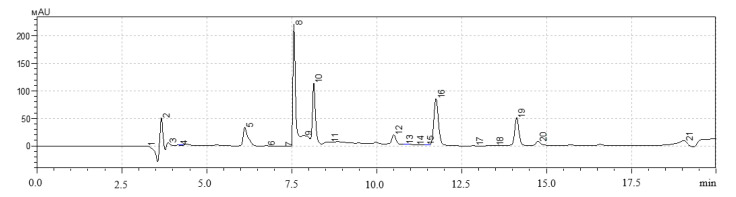
Chromatogram of extract No. 2 of *H. neglectum* hairy roots (300 nm): peak 8—gallic acid, 10—quercetin, 12—protocatechic acid, 16—chlorogenic acid, 19—caffeic acid.

**Figure 10:**
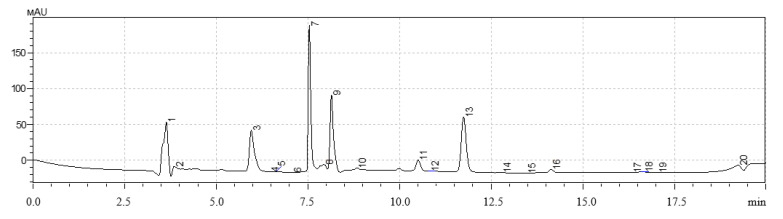
Chromatogram of extract No. 3 of *H. neglectum* hairy roots (300 nm): peak: 7—gallic acid, 9—quercetin, 11—protocatechic acid, 13—chlorogenic acid, 16—caffeic acid.

The results of the evaluation of the qualitative and quantitative composition of polyphenols in the studied extract samples of *H. neglectum* hairy roots are shown in Figure 8, Figure 9 and Figure 10 and in Table 8, Table 9 and Table 10.

The dominant polyphenols in the studied samples of extracts of *H. neglectum* hairy roots were determined using the HPLC method: gallic acid, quercetin, and protocatechic acid. It was found that the content of the target BAS, quercetin, in sample No. 1 was on average 2.1 times higher than in samples No. 2 and 3. Consequently, the following extraction parameters are optimal for the accumulation of quercetin: extractant—50% water–ethanol solution, hydromodulus—40:1, temperature 60 ± 2 °C, duration 1 h.

### 3.3. Results of Isolation and Purification of Target Metabolites

**Figure 11:**
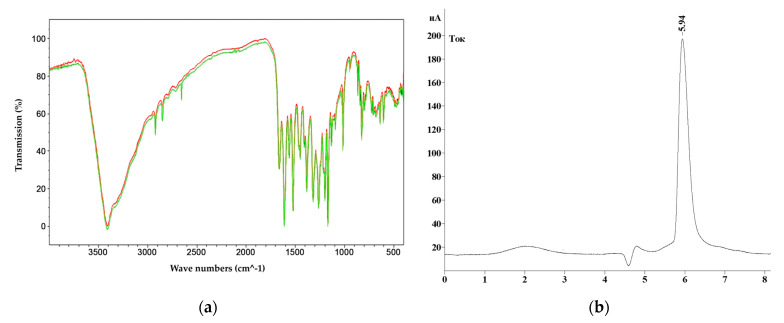
Results of identification of the isolated quercetin: (**a**) IR-spectrum (red color—spectrum of the reference sample of quercetin (98%); green color—spectrum of the investigated quercetin (95%)); (**b**) HPL-chromatogram.

Quercetin was isolated and purified using chromatography from extracts of hairy roots with the highest AOA (purification degree not less than 95%). The results of the identification of isolated individual substance from extracts of hairy roots are presented in Figure 11.

The given infrared spectra coincide with the absorption bands of the standards.

### 3.4. Results of Studying the Bioactivity of Quercetin Solutions

**Figure 12:**
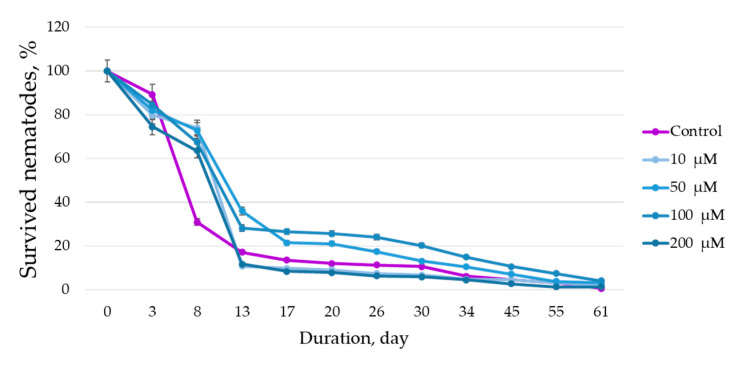
Effect of quercetin solutions on the life span of nematodes.

**Figure 13:**
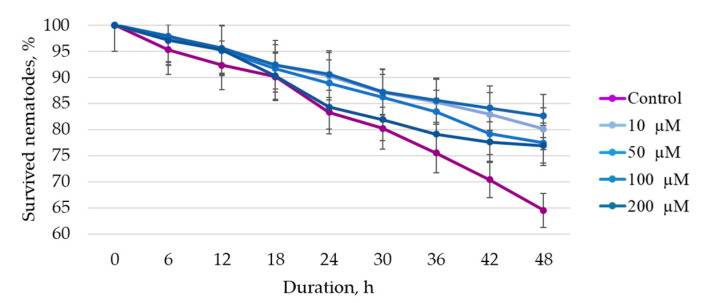
Effect of quercetin solutions on nematode survival under oxidative stress.

To assess the bioactivity of the target BAS, stock solutions in DMSO at a concentration of 10 mM were prepared. To assess the effect of BAS on the model organism *C. elegans*, solutions with concentrations of 2000 μM, 1000 μM, 500 μM, and 100 μM (in distilled water) were prepared. Figure 12 shows the results of the effects of the studied BAS in different concentrations on the life span of nematodes and Figure 13 shows the survival under oxidative stress.

The results showed that all quercetin solutions increased the lifespan of the nematodes. The best results were obtained on the 17th day with the 100 μM solution of quercetin, which on the 61st day increased the nematode life span by a factor of 2.06 when compared to the other solutions and by a factor of 6 when compared to the control. The data obtained do not contradict the data reported in the scientific literature [53]. For example, K. Pallauf et al. [54] demonstrated that quercetin in concentrations from 70 to 200 μM prolonged the life span of nematodes in seven of nine studies.

The results demonstrate that the survival rate was higher when 100 µM quercetin were added compared to the control and other samples at all study time points. For example, at 48 h of the experiment, the percentage of surviving nematodes was 1.30 times higher compared to the control, and on average 1.05 times higher compared to other samples. The obtained data do not contradict the literature data indicating that the quercetin solution isolated from plant raw materials can reduce the negative effect of oxidative stress, i.e., increase the percentage of surviving nematodes [53,54,55]. However, the scientific literature indicates that quercetin in conditions where the organism has a high level of oxidative stress increases damage to nematodes [54].

**Figure 14:**
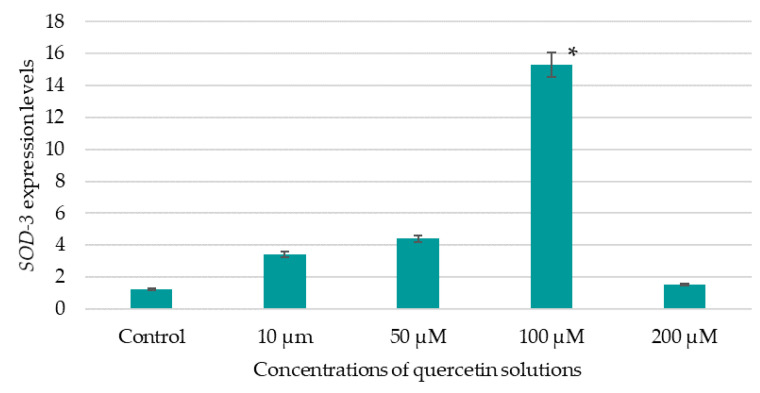
Effect of the studied quercetin solutions on *SOD-3* expression; data are presented as mean value ± SD (n = 3); * are values significantly different from the others (*p* < 0.05), according to the Tukey post hoc criterion score.

Figure 14 shows the results of evaluating the effect of the studied BAS solutions of different concentrations on the expression of *SOD-3*.

The data obtained indicate that a 100 µM quercetin solution led to an increase in *SOD-3* expression by a factor of about 12.5 compared to the control (expression value 1.22). The scientific literature provides evidence that quercetin isolated from plant objects exhibits the ability to influence *SOD-3* expression [13]. A study by B. Ayuda-Durán et al. [56] showed that quercetin at a concentration of 200 µM had a positive effect on the survival of nematodes under oxidative stress. However, compared to controls, quercetin solution at a concentration of 200 µM had the least impact on *SOD-3* expression in the studies performed by the authors. In the present study, quercetin at concentrations from 10 μM to 100 μM showed the ability to increase the percentage of surviving nematodes under oxidative stress. To obtain more accurate results when assessing the effects of quercetin on a model organism, analyses on more complex model objects, such as rodents, are required.

**Figure 15:**
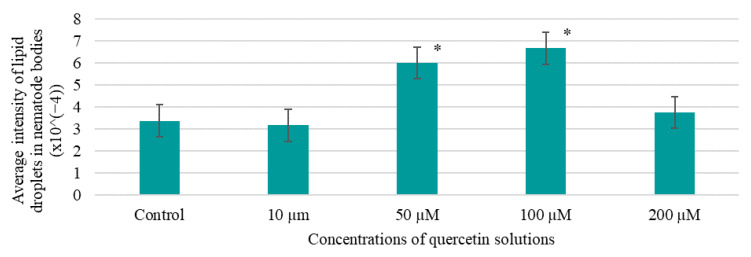
Effect of the studied BAS solutions on the accumulation of lipid inclusions in nematode bodies; data are presented as mean value ± SD (n = 3); * are values significantly different from the others (*p* < 0.05), according to the Tukey post hoc criterion score.

The results of the effect of quercetin solutions on changes in lipid inclusions in nematode bodies are shown in Figure 15.

The results demonstrate that quercetin solutions with concentrations from 50 to 200 μM had no activity in reducing the accumulation of lipid fractions in the nematode bodies. The best activity was shown by the 10 µM quercetin solution—the accumulation of lipid fractions was 1.06 times lower compared to the control. In the literature review, no publications on the effect of quercetin on the accumulation of lipid fractions in nematode bodies were found.

A 1.0 M stock solution of quercetin in 70% aqueous ethanol solution was prepared to evaluate AOA. Quercetin solutions were prepared by dilution in water at concentrations of 1000 μM, 800 μM, 600 μM, 400 μM, and 200 μM. The AOA of quercetin solutions are shown in Table 11.

In the course of the study, it was found that quercetin solutions (solvent: 70.0% water–ethanol mixture) of various concentrations exhibited an AOA that was similar to that of the control. The AOA of quercetin solutions was 1.7 times higher on average than the activity of vitamin C (control). The obtained data also confirm that the ABTS–radical scavenging activity of the solutions increased as the concentration of BAS in them increased, which does not contradict the literature data. The data obtained do not contradict the data reported in the scientific literature and once again confirm that quercetin, due to its structure, namely, the presence of four hydroxyl groups, exhibited a high ability to remove free radicals [12,13,14].

## 4. Discussion

Atherosclerosis is a chronic inflammatory disease associated with the accumulation of lipids in the aortic wall and the formation of foam cells, leading to the blockage of blood vessels, which is the cause of CVDs [57]. CVDs are a serious problem in modern society, as they are one of the main causes of death in the population. Prevention of AD through the control of the main risk factors for its development is an important step in modern healthcare. Prevention of atherosclerosis is possible due to the systematic use of BAS–cardioprotectors. Promising BAS–cardioprotectors are polyphenols (quercetin, baicalin, chlorogenic acid, curcumin, etc.) [5]. A promising source of BAS–cardioprotectors are, for example, plants used in traditional medicine in various countries: *Scutellaria baicalensis* Georgi. [58], *Curcuma longa* [59], etc.

It has been proven that quercetin exhibits anti-atherosclerotic potential in vitro and in vivo (on a model organism, *C. elegans*). The results obtained do not contradict the literature data. According to F. Surco-Laos et al. [60], quercetin and its derivatives provide antioxidant protection to *C. elegans*, increasing nematode resistance to the influence of stress factors. The survival rate of nematodes treated with 200 µM quercetin under oxidative stress was 12% higher than that of the control. In this study, the addition of 100 µM quercetin increased survival by 7% when compared to controls. However, the study conditions were different: F. Surco-Laos et al. [60] used juglone to create stress conditions, whereas our research involved paraquat.

B. Ayuda-Durán et al. [56] proved that increased resistance of *C. elegans* to oxidative stress conditions is associated with the ability of quercetin to influence insulin/insulin-like growth factor 1 (IGF-1) signaling pathway (IIS) and the expression of some genes related to stress response. According to A. Kampkötter et al. [53], quercetin suppressed the expression of the *SOD-3* gene, which contradicts the data obtained by the authors. In this study, it was proven that quercetin, which exhibits high antioxidant activity, can increase the expression of *SOD-3* and the survival of nematodes under conditions of oxidative stress (the expression of *SOD-3* was about 12.5 times higher compared to the control). Therefore, more research is required, for example, with juglone.

The literature review failed to find publications that describe the effect of quercetin on the accumulation of lipid fractions in nematodes. However, data presented by X. Qin et al. [61] show that rutin can reduce the accumulation of lipid fractions in nematodes by influencing the expression of genes involved in lipid metabolism (*fat-7*, *acox-1.3*, *stdh-3*). Rutin is a glycoconjugate composed of quercetin and rhamnose–glucose disaccharide; thus, it may be possible to use quercetin to reduce the levels of lipid fractions in other model objects in the future.

The results obtained in this research contribute to the overall study of the bioactivity of quercetin as a promising substance for the prevention of atherosclerosis and, therefore, CVDs.

## 5. Conclusions

The hairy roots of *H. neglectum* were discovered to be a promising source of quercetin, which has a variety of bioactive properties, such as antioxidant activity in vitro, a positive effect on longevity, survival under oxidative stress conditions, SOD-3 gene expression, and a reduction in lipid inclusion accumulation in vivo in *C. elegans*. Therefore, quercetin isolated from the hairy roots of *H. neglectum* is suitable for use as a component of dietary supplements with preventive action (anti-atherosclerotic potential, cardioprotective potential, and geroprotective effect).

More research is required to assess the anti-atherosclerotic potential of quercetin isolated from *H. neglectum* hairy roots, for example, using cell models such as THP-1 human monocyte cells, EA.hy926, and Huh7 liver hepatoma cell culture [5]. Furthermore, the presence or absence of cytotoxic activity of quercetin using HEP-G2 cells, cytotoxic activity against HEP-G2 cells [62,63], and other factors need to be investigated. The anti-atherosclerotic mechanism of action of quercetin on various model objects in vitro and in vivo should be studied.

## Figures and Tables

**Table 1:** Composition of nutrient media used for the cultivation of *H. neglectum* hairy roots (composition per 1 dm^3^).

Components	Nutrient Medium			
	No. 1	No. 2	No. 3	No. 4 (Control)
Major salts B5, 20×, cm^3^	50.0	50.0	50.0	50.0
Minor salts B5, 20×, cm^3^	10.0	10.0	10.0	10.0
Fe-EDTA, cm^3^	5.0	5.0	5.0	5.0
Thiamine, mg	0.1	0.1	0.1	0.1
Pyridoxine, mg	0.1	0.1	0.1	0.1
Nicotinic acid, mg	0.5	0.5	0.5	0.5
Sucrose, g	30.0	30.0	30.0	30.0
NAA, mg	1.0	2.0	–	–
6–BAP, mg	0.5	1.0	1.5	–
Agar, g	7.0	7.0	7.0	7.0

NAA—alpha-naphthylacetic acid, 6-BAP—6-benzylaminopurine.

**Table 2:** Amplification parameters.

Primer No. 1	5′-ATGGATCCCAAATTGCTATTCCTTCCACGA-3′
Primer No. 2	5′-TTAGGCTTCTTTCTTCAGGTTTACTGCAGC-3′
Parameters	Denaturation 3 min at 94 °C, 30 amplification cycles: 50 s melting at 94 °C, 1 min annealing at 53 °C, 1 min elongation at 72 °C, final elongation for 10 min at 72 °C

**Table 3:** Extraction parameters of the *H. neglectum* raw material.

No.	Extraction Parameters				Reference
	Extractant	Hydromodule	Temperature	Duration	
1	50% ethanol	40:1	60 ± 2 °C	1 h	[41,42,43]
2	60% ethanol				
3	70% ethanol				

**Table 4:** RNA amplification steps (steps 2–4 repeated 39 times).

Step	Name	Settings
1	Pre-denaturation	95 °C, 5 min
2	Denaturation	95 °C, 30 s
3	Annealing	61 °C, 30 s
4	Elongation	72 °C, 30 s
5	Arresting a reaction	65 °C, 5 s

**Table 5:** Hairy roots cultivated on various NM.

NM	Total Number of Explants, pcs	% of Explants That Formed Roots	Root Length, cm	Start of the Stationary Phase, Day
No. 1	30.5 ± 1.0	65.0 ± 1.0	1.32 ± 0.05	28.0 ± 1.0
No. 2	30.0 ± 1.0	65.0 ± 1.0	1.35 ± 0.05	
No. 3	32.0 ± 1.0	70.0 ± 1.0	1.39 ± 0.05	
No. 4	31.5 ± 1.0	68.5 ± 0.5	1.35 ± 0.02	

Data are presented as mean value ± SD (n = 3).

**Table 6:** Extraction parameters selected during the literature review for biomass of hairy roots.

No.	Sample Weight, g	Extractant		Hydromodule	Temperature	Duration	Other
1	1.004 ± 0.001	Aqueous ethanol solution	50%	40:1	60 ± 2 °C	1 h	15 min of ultrasonic treatment followed by extraction
2	1.016 ± 0.003		60%				
3	1.018 ± 0.002		70%				

Data are presented as mean value ± SD (n = 3).

**Table 7:** AOA of hairy root extracts obtained at the selected extraction parameters, %.

Hairy Roots	Samples		
*H. neglectum*	No. 1	No. 2	No. 3
	94.07 ± 0.01	94.40 ± 0.01	94.30 ± 0.01

Data are presented as mean value ± SD (n = 3).

**Table 8:** Component composition of the major polyphenols of sample No. 1.

Peak No.	Retention Time, Minutes	Component	Quantitative Content, mg/g
5	7.617	Gallic acid	5.15 ± 0.04
8	8.298	Quercetin	4.32 ± 0.05 *
9	10.497	Protocatechic acid	0.08 ± 0.01
10	11.894	Chlorogenic acid	0.09 ± 0.07
11	14.138	Caffeic acid	0.01 ± 0.01

Data are presented as mean value ± SD (n = 3). * are values significantly different from the others (*p* < 0.05), according to the Tukey post hoc criterion score.

**Table 9:** Component composition of the major polyphenols of sample No. 2.

Peak No.	Retention Time, Minutes	Component	Quantitative Content, mg/g
5	7.565	Gallic acid	3.07 ± 0.04
8	8.150	Quercetin	2.94 ± 0.04
9	10.507	Protocatechic acid	0.07 ± 0.01
10	11.747	Chlorogenic acid	0.10 ± 0.01
19	14.124	Caffeic acid	0.03 ± 0.00

Data are presented as mean value ± SD (n = 3).

**Table 10:** Component composition of the major polyphenols of sample No. 3.

Peak No.	Retention Time, Minutes	Component	Quantitative Content, mg/g
5	7.540	Gallic acid	2.77 ± 0.03
8	8.143	Quercetin	1.64 ± 0.04
9	10.511	Protocatechic acid	0.08 ± 0.02
10	11.750	Chlorogenic acid	0.10 ± 0.06
16	14.138	Caffeic acid	0.02 ± 0.00

Data are presented as mean value ± SD (n = 3).

**Table 11:** AOA of quercetin solutions.

Substance	Solution Concentration				
	1000 μM	800 μM	600 μM	400 μM	200 μM
Quercetin	67.69 ± 2.59 *	66.43 ± 2.33 *	57.38 ± 2.28 *	49.44 ± 2.04 *	29.53 ± 1.45 *
Vitamin C (control)	50.89 ± 1.59	40.03 ± 1.23	37.28 ± 1.55	19.67 ± 0.57	18.16 ± 0.56

Data are presented as mean value ± SD (n = 3); * are values significantly different from the others (*p* < 0.05), according to the Tukey post hoc criterion score.
